# Unpacking the complexities of de-implementing inappropriate health interventions

**DOI:** 10.1186/s13012-019-0960-9

**Published:** 2020-01-09

**Authors:** Wynne E. Norton, David A. Chambers

**Affiliations:** Division of Cancer Control and Population Sciences, National Cancer Institute, 9609 Medical Center Drive, #3E424, Bethesda, MD 20850 USA

**Keywords:** De-implementation, Ineffective, Low-value, Unproven, Harmful, De-adoption, De-escalation, Overtreatment, Overscreening, Overuse, Implementation science, Medical reversal

## Abstract

De-implementing inappropriate health interventions is essential for minimizing patient harm, maximizing efficient use of resources, and improving population health. Research on de-implementation has expanded in recent years as it cuts across types of interventions, patient populations, health conditions, and delivery settings. This commentary explores unique aspects of de-implementing inappropriate interventions that differentiate it from implementing evidence-based interventions, including multi-level factors, types of action, strategies for de-implementation, outcomes, and unintended negative consequences. We highlight opportunities to continue to advance research on the de-implementation of inappropriate interventions in health care and public health.

## Background

De-implementing inappropriate health interventions is essential for improving population health, maintaining public trust, minimizing patient harm, and reducing unnecessary waste in health care and public health. In recent years, researchers, health professionals, funders, policymakers, and patients have become increasingly focused on the need to stop or reduce the use of inappropriate health interventions. This has been spurred in part by empirical data on overuse of health interventions, identification of hundreds of medical reversals, all-too-common use of untested treatments, and prevalence of low-value healthcare services [1–3]. Interest in this area is reflected in international campaigns [4], conferences [5], research and professional networks [6–8], journals [9], and funding announcements [10], as well.

Discussion of de-implementation of ineffective, contradicted, mixed, and untested health interventions (collectively referred to as “inappropriate” for sake of brevity unless otherwise indicated) is increasingly prominent in the published literature. A scoping review identified 43 different terms for de-adoption (e.g., de-prescribe, abandon, de-implement) [11], and several models and frameworks for conceptualizing, understanding, and guiding de-implementation have been proposed [12–15]. Barriers to de-implementation have been explored, and effective (albeit few) strategies to help drive de-implementation have been identified through rigorous trials [16].

To complement ongoing efforts, this commentary seeks to unpack de-implementation by taking a more nuanced look at multi-level factors, actions, strategies, outcomes, and unintended consequences. This examination is informed by a review of the literature, comparison to and reflection on implementation of evidence-based interventions, and a recently published de-implementation framework [12]. We highlight key areas for future research and encourage the field to consider seemingly minor yet critically important distinctions between the implementation of new, evidence-based interventions and the de-implementation of currently delivered inappropriate interventions.

To this end, we first examine multi-level factors influencing de-implementation. These include characteristics of (1) the currently delivered inappropriate health intervention, program, guideline, treatment, or test (collectively referred to as “intervention”) to be de-implemented; (2) the patient (i.e., individual who receives the intervention); (3) the health professional (e.g., public health practitioner or healthcare provider who delivers the intervention); and (4) the organization (e.g., health system or clinic in which or through which the intervention is delivered). We highlight aspects within each of these factors, discuss their role in hindering de-implementation, and describe how they may be slightly or significantly different from the implementation of new, evidence-based interventions. Second, we describe four types of action for de-implementation (e.g., remove, replace, reduce, and restrict) and a range of strategies for de-implementation. Third, we discuss the complexity of defining and measuring optimal outcomes of de-implementation, and the unintended, negative consequences that may arise from achieving such outcomes. We close by highlighting near-term opportunities for researchers to broaden and deepen our understanding of de-implementation.

## Multi-level factors affecting de-implementation

As with the implementation of new, evidence-based interventions, factors affecting the de-implementation of currently delivered, inappropriate interventions are multi-level, complex, and context specific. The multiple levels at which de-implementation is affected—including the inappropriate health intervention, patient, health professional, and organization—overlap with those that affect implementation, but likely operate in ways that are relatively unique.

### Health intervention characteristics

As with implementation, characteristics of the health intervention undoubtedly affect de-implementation. These features likely include those first articulated by Rogers: relative advantage, compatibility, complexity, trialability, and observability [17], as well as costs, adaptability, form, risks, and interdependence [18, 19]. The extent to which intervention characteristics affect de-implementation with the same magnitude and in the same direction as they do for implementation, however, is yet to be understood. There are two characteristics of an inappropriate intervention that may be particularly unique to de-implementation, including strength of evidence and level of complexity.

The strength of evidence of a candidate intervention for implementation is ideally one that has strong, empirical data that it improves proximal or distal patient health outcomes or behaviors; demonstrated to be efficacious, effective, and cost effective (or even cost saving); and shown to have a reasonable effect size and number needed to treat [20, 21]. The minimum strength of evidence needed to warrant implementation, however, has largely been overlooked by the field to date; instead, interventions have been loosely characterized dichotomously as either evidence-based or non-evidence-based. Only recently has the field begun to explore how gradations in the strength of evidence of a new intervention affect implementation, or consider whether implementation should occur at all.

This emerging line of thinking in implementation research should be extended and applied to de-implementation research. In an effort to help move this forward, we propose a classification system of interventions for de-implementation that vary by the strength of evidence. These include interventions that are *ineffective*, *contradicted*, *mixed*, or *untested*. *Ineffective* interventions are those for which a few (if not many) high-quality studies have shown to not improve patients’ health outcomes or behaviors and may actually incur more harm than benefit. *Contradicted* interventions (otherwise known as medical reversals [22]) are those for which a newer, higher-quality study (or studies) indicates that the health intervention does not improve outcomes, which is contrast to a previous, lower-quality study (or studies) indicating that it does work. *Mixed* interventions are those for which the quantity and quality of evidence in support of and against the effectiveness of the intervention is approximately equal. Finally, *untested* interventions are those for which little to no empirical evidence exists about their effectiveness because they have yet to be studied. Although subtle, these distinctions are important, as they are likely associated with distinct multi-level barriers to de-implementation and the selection and use of tailored strategies for de-implementation. Understanding how the strength of evidence of a currently delivered, inappropriate intervention affects the de-implementation process can help identify and prioritize what, when, how, and to what extent de-implementation can or should occur.

The complexity of a health intervention also plays an important role in de-implementation. Relatively simple interventions, such as medications or tests, may be easier to de-implement and require fewer and less intense strategies compared to more complex interventions, such as surgical procedures or bundles of interventions delivered across the care continuum. Replacing an existing, inappropriate intervention with a new, evidence-based intervention may be particularly challenging if the latter requires additional staff, resources, time, and acquisition of new skills in comparison to the former. This may be even more difficult if the inappropriate intervention has been delivered for a long period of time or if the new intervention requires learning complex, technical skills that are contradictory in form, function, or philosophy to those required by the existing, inappropriate intervention.

### Patient characteristics

As with implementing a new, evidence-based intervention, patient-level factors that affect de-implementation include attitudes, behavioral skills, social norms, and demographic characteristics. Compared to implementation, however, there are three patient-level factors that are relatively unique to de-implementation: fear and anxiety, inaccurate perceptions about health interventions and health care, and lack of trust in health care and public health establishments. These areas are ripe for research and have important implications for how best to develop, select, and test barrier-specific strategies for de-implementation.

Patients are often reluctant to forego additional yet unnecessary screenings or diagnostic tests for fear of being diagnosed too late to benefit from available treatment. Patients may also have anxiety over perceived (yet inaccurate) susceptibility to a disease or over not knowing one’s health status. In both circumstances, they may prefer to receive a health intervention to reduce their anxiety, even if it is of low value or has poor predictive validity. Inaccurate yet pervasive personal beliefs and social norms, such as more care is better care or newer health technologies are better than older ones, can further hinder de-implementation efforts. Distrust of the medical establishment, coupled with media coverage showcasing conflicting health information, contributes to patients’ lack of confidence in health professionals and the healthcare enterprise. Moreover, de-implementing an existing health intervention—particularly one that a patient has received often and long-term—has the potential to damage the patient-provider relationship and (inaccurately) be perceived by patients as health professionals’ withholding necessary care.

### Health professional characteristics

Characteristics of health professionals that affect de-implementation again overlap with some of those for implementation, such as behavioral skills, self-efficacy, and knowledge. Characteristics unique to de-implementation, however, include health professionals’ past experience of negative events, cognitive dissonance, and fear of medical malpractice.

Health professionals may be particularly reluctant to de-implement an intervention in the future if doing so led to negative consequences in the past. This would be applicable to health professionals who have been wrongly accused of withholding or rationing care, reprimanded for their care decisions, or have had patients experience severe, debilitating health outcomes—including death—that resulted from the appropriate de-implementation of an intervention, but one that was nonetheless perceived by patients, family members, colleagues, or supervisors to be in error. Cognitive dissonance is another barrier toward de-implementation, as it creates an undesirable state of tension stemming from the discrepancy between one’s belief in providing high-quality care and the delivery of an inappropriate (or even harmful) intervention. Health professionals may effectively avoid this state of dissonance by resisting de-implementation efforts. Fear of medical malpractice lawsuits, which can damage health professionals’ reputation and increase malpractice premiums, is a substantial barrier to de-implementation, and particularly so for low-value interventions [23–26]. Research is needed to understand the contextual factors that may cue the practice of defensive medicine, identify when it is most likely to occur, and develop strategies to prevent or reduce its occurrence when it results in unnecessary care.

### Organizational characteristics

As with implementation, characteristics of the organization that affect de-implementation include organizational culture and climate, leadership, resources, and structure. Although some organizations may welcome the opportunity to de-implement inappropriate interventions to the extent that it affords them an opportunity to improve efficiency, optimize use of limited resources, and reduce burden, other organizations may resist. Some organizations may be less likely to remove an inappropriate intervention if it generates considerable revenue or if it prevents them from showcasing an innovative (albeit unproven or low-value) intervention that gives them a competitive edge over other organizations. Organizations may also resist de-implementing interventions that have a greater return-on-investment or revenue-generating reimbursement structure, or among specialty health practices where health professionals may have fewer revenue streams. Organizations may resist supporting a culture of de-implementation for fear of liability. This may be particularly pronounced when it comes to reducing the frequency or intensity of delivering low-value interventions, for which it is less clear or even controversial to whom and when it would be considered low- value care.

## Types of action and tailored strategies for de-implementation

### Types of action for de-implementation

The type of action involved in implementation generally includes some variation of starting and/or increasing the use of an evidence-based intervention. While important, a more granular conceptualization of implementation actions may be helpful, to the extent that strategies for one type of action (e.g., initiating) may be different than strategies for another (e.g., increasing). We propose four types of actions that may occur under the broad concept of de-implementation. Although subtle, each action likely differentiates itself with respect to multi-level factors, strategies, outcomes, and unintended negative consequences. For these reasons, teasing apart the type of action involved in de-implementation is crucial.

De-implementation may involve removing, replacing, reducing, or restricting the delivery of an inappropriate intervention. *Removing* an intervention is the process of stopping the delivery of an inappropriate intervention entirely. Examples include removal of a drug from the market or recall of a device. *Replacing* an intervention involves stopping an inappropriate intervention and starting a new, evidence-based intervention that targets the same or similar proximal or distal patient-level health behaviors or health outcomes. Examples include replacing opioid prescriptions as first-line therapy for treatment of acute lower back pain with a stepped care approach, starting with physical therapy. *Reducing* an intervention involves changing the frequency and/or intensity with which that intervention is delivered. Examples include reducing the frequency with which screening tests are delivered (e.g., every 5 years instead of 3), reducing the intensity of medication dosage (e.g., 500 mg to 100 mg), or even a combination of both. Finally, *restricting* an intervention occurs when the scope of an intervention is narrowed by target population, health professional, and/or delivery setting. Examples of restriction include a change from universal to high-risk screening for patients, administration of a diagnostic test by primary care professionals and nurse practitioners to only primary care professionals, or treatment provided in both general and specialty clinics to only specialty clinics. Importantly, the intervention continues to be delivered—even at the same frequency and/or intensity—but is limited to a smaller or more targeted subset of patients, health professionals, and/or delivery settings. Each overarching action (i.e., remove, replace, reduce, or restrict) likely involves discrete processes and is comprised of a collection and sequence of different behaviors in pursuit of the overall action-specific outcome.

### Tailored strategies for de-implementation

As with implementation, multi-level strategies for de-implementation should be developed and tested to be context- and barrier-specific but feasible, adaptable, and generalizable to other settings, where appropriate. Some strategies, such as stakeholder engagement, leadership buy-in, and organizational readiness, are likely to be effective (and arguably necessary) for both implementation and de-implementation, whereas others may only be applicable to de-implementation. Unique strategies for de-implementation may include those that target the unique barriers to de-implementation. These might include affective-based approaches to attenuate patients’ anxiety over missing a diagnosis, medical malpractice tort reform to reduce health professionals’ fear of litigation, and financial disincentives for organizations to use ineffective or unproven interventions. Research is needed to test whether these and other barrier-specific strategies for de-implementation are effective and to understand if or how their effectiveness varies by context.

Multi-level strategies for de-implementation should also match the target action for de-implementation, as different actions are underpinned by different theories, frameworks, and models for change. Theories of habit formation and disruption [27] suggest that the most effective way to reduce the use of an inappropriate intervention may be to change the context and environmental cues, particularly so when the intervention is simple and requires less cognitive or behavioral effort. For example, effective strategies for ordering fewer lab tests may include changing order sets in the EHR system (disrupting environmental cues) and/or requiring written authorization (increasing cognitive and behavioral effort). Informed by individual and organizational theories of learning and unlearning, the most effective strategies for replacing an inappropriate intervention with a new, evidence-based intervention may include behavioral skills training, audit and feedback, and leadership support [13, 28–30].

Future research on de-implementation should explore how to identify multi-level barriers, match them with appropriate strategies, and calibrate the barrier-strategy pairing as it changes over time. One approach for doing so would be to assess multi-level barriers over time and leverage those data to select and deploy barrier-specific strategies for de-implementation. Similar in concept to diagnostic measures, ongoing assessments at pre-determined intervals throughout the de-implementation process would help “diagnose” time-varying barriers and “treat” them with multi-level strategies for de-implementation. In doing so, researchers would be able to identify strategies that are no longer needed, new strategies that should be deployed, and current strategies that should be sustained to achieve target outcomes. In addition, this data-driven approach would allow for testing theory-based hypotheses; identifying longitudinal moderators, single- and multi-level mediators, and mechanisms of de-implementation; and assessing how the relationship between barriers and strategies changes over time [31–35]. Rapid, state-of-the-art qualitative methods [36] would complement these quantitative data and provide a more in-depth understanding of context and process.

## Outcomes of de-implementation and unintended negative consequences

### Outcomes of de-implementation

Outcomes of de-implementation should reflect the type of action for de-implementation and the time frame in which those outcomes should be achieved. Data sources may include self-report, claims data, policy or procedural changes, and/or short- and long-term patient health outcomes. Outcomes of de-implementation should also include changes in multi-level barriers that are the target of strategies and account for how they may fluctuate over time.

Analogous to the time horizon around implementing evidence-based interventions, identifying and defining “successful” or “optimal” outcomes of de-implementation is complicated by the duration and pace at which de-implementation can or should occur. When, how quickly, and to what extent an intervention should be de-implemented varies as a function of characteristics of the intervention (e.g., strength of evidence, cost-effectiveness) and the magnitude of the problem (e.g., harm, prevalence) that the intervention incurs. Indeed, some interventions should be removed as quickly as possible, reduced over an extended period of time, or follow a sequence of actions for which the duration should vary (e.g., gradually reduce over 6 months and then remove within 1 month). The time frame and pace at which de-implementation should occur can be unspecified or unimportant, too. However, without considering time-to-de-implementation, one may inadvertently increase the potential for harm by de-implementing an intervention too quickly or not quickly enough. Research is needed to understand how to determine optimal rates for de-implementation, how quickly different strategies for de-implementation are able to meet target outcomes, and at what cost.

### Unintended negative consequences

Even when successful, de-implementing an inappropriate health intervention may increase the probability of unintended negative consequences for patients, health professionals, and organizations. For example, one optimal outcome of de-implementation may be cessation of an inappropriate screening test to patients. Achieving this outcome, however, could lead to a decrease in patients’ trust in the healthcare system and subsequently lead to poorer engagement in care and missed opportunities for detecting diseases for which the patient is at high-risk. Health professionals may successfully reduce the frequency with which they administer an inappropriate intervention, but compensate for this change by increasing the use of another intervention downstream, intentionally or otherwise. Organizations may need to downsize if they replace a time-intensive, costly intervention with a more efficient one; this, in turn, could inadvertently lower employees’ trust in the organization and increase staff turnover. Successful de-implementation outcomes at one level may also lead to negative consequences at another level. For example, although patients may benefit from stopping an inappropriate medication, organizations may be harmed if they lose revenue and may even be less likely to de-implement an intervention in the future. Research is needed to understand the full range of short- and long-term unintended negative consequences of de-implementation and to develop and test approaches to mitigate or prevent their occurrence.

## Opportunities for advancing research on de-implementation

Opportunities exist to broaden and deepen our scientific understanding of de-implementation. For example, investigators with current or forthcoming implementation trials that involve replacing an inappropriate intervention with an evidence-based intervention (that targets the same or similar health outcomes) could explore how this process unfolds. Researchers could collect qualitative data from key decision-makers to better understand why one intervention is being replaced by another, and explore how the decision-making process is informed by differences between the interventions in terms of strength of evidence, complexity, and resource requirements. Monitoring adaptations to the new, evidence-based intervention may be particularly important, to the extent that the new intervention may drift toward and increasingly resemble the old, inappropriate intervention, and subsequently require more intense strategies to redirect toward more appropriate adaptations.

Existing databases (e.g., Centers for Medicare and Medicaid Services, Agency for Healthcare Research and Quality’s Healthcare Cost and Utilization Project) can be mined to help jumpstart the field. For example, researchers could take advantage of natural experiments [37, 38] and use a controlled interrupted time series design to examine the effect of a change in health policy, insurance coverage, guideline rating, or federal approval or clearance on the de-implementation of inappropriate interventions. Following the approach used by the Dartmouth Atlas Project [39] and the National Health Service Atlas of Variation [40], mapping variation in de-implementation may be particularly useful, to the extent that it can identify positive deviants that de-implement rapidly, geographic regions for which de-implementation lags, and the types of interventions and health conditions for which de-implementation does not occur at all. Process evaluation can help better understand the context in which variation in de-implementation occurs [41].

## Conclusion

Acknowledging and unpacking the complexities of de-implementation helps support more and better research in this area. The issues discussed herein (summarized in Table 1) are a collection of concepts that make de-implementing currently delivered, inappropriate interventions relatively distinct from implementing new, evidence-based interventions. Ultimately, a more nuanced understanding of the context in which de-implementation occurs provides a greater opportunity for minimizing harm to patients, maximizing efficient use of resources, and improving the overall health of populations.

**Table 1:** Overview of complexities of de-implementation and sample research questions

Multi-level factors	Characteristics	Sample research questions
Intervention	Strength of evidence	What happens if the strength of the evidence for an intervention changes during a de-implementation trial?
	Complexity	Are simpler interventions easier to de-implement than more complex interventions?
Patient	Anxiety, fear, and worry	What are some predictors of patients’ level of anxiety in anticipation of no longer receiving an intervention?
	Inaccurate beliefs and social norms	What are some common misperceptions about de-implementation among patients?
	Distrust of medical establishment	Under what conditions might de-implementation lead to patients’ distrust of health professionals?
Health professional	Negative past events	What is the relationship between severity of negative past events, frequency of negative past events, and health professionals’ willingness to de-implement?
	Cognitive dissonance	What are some predictors of health professionals who experience cognitive dissonance?
	Fear of medical malpractice	What differentiates health professionals who fear medical malpractice and engage in defensive medicine from those who do not?
Organization	Revenue	Why do some organizations embrace the de-implementation of revenue-generating interventions whereas others resist?
	Competitive advantage	Who is involved in making decisions to market an intervention for which the strength of the evidence is mixed, and how are those decisions made?
	Liability	Is there a liability threshold above which organizations are less likely to de-implement an intervention?
Types of action	Description	Sample research questions
Remove	Stop delivering an inappropriate intervention	How does one determine the pace at which an intervention should be removed?
Replace	Replace a currently delivered inappropriate intervention with a new, evidence-based intervention targeting the same or similar patient outcomes	What are the minimum criteria for deciding when to replace one intervention with another?
Reduce	Reduce (frequency and/or intensity) use of an inappropriate intervention	Is it more difficult to reduce both the frequency and intensity of an intervention versus only the frequency or intensity of an intervention?
Restrict	Narrow to whom, by whom, and/or where the intervention is delivered	What are some of the unintended negative consequences of restricting the delivery setting in which an intervention is delivered?
Multi-level targets	Potential strategies	Sample research questions
Patient	Affective-based interventions to reduce anxiety, fear, and worry	What role can caregivers play in reducing patients’ fear of missing a diagnosis?
Health professional	Medical malpractice tort reform	Can medical malpractice tort reform reduce defensive medicine? Is tort reform more effective in some specialties or for some types of interventions than others?
Organization	Identify alternative sources of revenue	What toolkits can help organizations identify alternative sources of revenue that will facilitate de-implementation?

Note: This is not a comprehensive list of all factors that affect de-implementation but rather a summary of those that may be particularly applicable or unique to de-implementation of inappropriate health interventions as compared to implementation of new, evidence-based health interventions

## Data Availability

Not applicable.
